# Invasive Chloroplast Population Genetics of *Mikania micrantha* in China: No Local Adaptation and Negative Correlation between Diversity and Geographic Distance

**DOI:** 10.3389/fpls.2016.01426

**Published:** 2016-09-21

**Authors:** Ting Wang, Zhen Wang, Guopei Chen, Chunbo Wang, Yingjuan Su

**Affiliations:** ^1^College of Life Sciences, South China Agricultural UniversityGuangzhou, China; ^2^College of Life Sciences, Nanjing Agricultural UniversityNanjing, China; ^3^School of Life Sciences, Sun Yat-sen UniversityGuangzhou, China; ^4^Research Institute of Sun Yat-sen UniversityShenzhen, China; ^5^Institute for Technology Research and Innovation, Sun Yat-sen UniversityZhuhai, China

**Keywords:** *Mikania micrantha*, invasive chloroplast population genetics, no local adaptation, negative correlation, diversity, geographic distance

## Abstract

Two fundamental questions on how invasive species are able to rapidly colonize novel habitat have emerged. One asks whether a negative correlation exists between the genetic diversity of invasive populations and their geographic distance from the origin of introduction. The other is whether selection on the chloroplast genome is important driver of adaptation to novel soil environments. Here, we addressed these questions in a study of the noxious invasive weed, *Mikania micrantha*, which has rapidly expanded in to southern China after being introduced to Hong Kong in 1884. Seven chloroplast simple sequence repeats (cpSSRs) were used to investigate population genetics in 28 populations of *M. micrantha*, which produced 39 loci. The soil compositions for these populations, including Mg abundance, were measured. The results showed that *M. micrantha* possessed relatively high cpSSR variation and differentiation among populations. Multiple diversity indices were quantified, and none was significantly correlated with distance from the origin of introduction. No evidence for “isolation by distance,” significant spatial structure, bottlenecks, nor linkage disequilibrium was detected. We also were unable to identify loci on the chloroplast genome that exhibited patterns of differentiation that would suggest adaptive evolution in response to soil attributes. Soil Mg had only a genome-wide effect instead of being a selective factor, which highlighted the association between Mg and the successful invasion. This study characterizes the role of the chloroplast genome of *M. micrantha* during its recent invasion of southern China.

## Introduction

*Mikania micrantha* H. B. K. (Asteraceae) represents one of the world’s 100 worst weeds (Holm et al., 1977). It is a perennial vine with both sexual and vegetative reproduction. The invasion history of *M. micrantha* in southern China can be clearly traced with voucher records. Its first appearance was documented at Hong Kong Zoological and Botanical Gardens in 1884 (Wang et al., 2003). After becoming naturalized by 1919, the weed started to spread at an alarming rate (Wang et al., 2003). In 1984, *M. micrantha* reached near Shenzhen (Wang et al., 2003), from where it rapidly spread throughout Guangdong, and then on to other southern and central China areas (Zhang L.Y. et al., 2004).

During colonization, invasive species are expected to experience sharp decreases in effective population size during founder events, which may have an important genetic impact (Dlugosch and Parker, 2008). Indeed, many invasive species are found to have reduced genetic variation in their introduced ranges (Dlugosch and Parker, 2008). In principle, a negative correlation is expected between the genetic diversity of a given invasive population and its distance from the origin of introduction in the invaded areas (Prugnolle et al., 2005). However, empirical results demonstrate that population genetic patterns can be complex and deviate from this expectation. For instance, a high level of genetic variation was revealed in the introduced populations of *M. micrantha* by using AFLP markers (Wang et al., 2012). Theoretical analyses have shown that factors, including high levels of growth rate, migration (gene flow), and dispersal distance, all can reduce the predicted effects of successive founder events and limit the loss of genetic diversity (Austerlitz et al., 1997). Besides, multiple introductions from a single or multiple source populations may also buffer the loss of genetic diversity (Dlugosch and Parker, 2008; Sakata et al., 2015). As the introduction of *M. micrantha* in southern China has an explicit origin, it provides an ideal subject to exam how genetic diversity evolves and is structured during the colonization process. Moreover, we have also conducted spatial autocorrelation analysis to estimate the spread potential of this invasive weed (Fitzpatrick et al., 2012).

Of diverse ecological factors that impact the population genetic patterns of invasive plants, soil is a crucial one that should be given special consideration. Soil provides essential nutrients for plants and has a decisive influence on their local adaptation (Hancock et al., 2011). Changes in soil metal concentrations therefore have the potential to drive local genetic adaptation (Alberto et al., 2010). Indeed, genetic differentiation in response to soil factors has been well documented in a wide range of plant taxa (Anacker et al., 2011; Misiewicz and Fine, 2014). *M. micrantha* has exhibited different soil preferences in invasive areas (Zhang L.Y. et al., 2004). Unlike in its native range, the weed grows on both dry soils and shady, more mesic sites in southern China (Zhang L.Y. et al., 2004), whose favorable soil moisture has consequently increased to higher than 15% (Huang et al., 2000). More importantly, the expansion of *M. micrantha* can also induce changes in the soil nutrient content, which in turn promotes growth (Liu et al., 2012). Therefore, the initial differences and subsequent changes of soil composition in invasive regions may act as selective factors, driving adaptive genetic differentiation. We have identified adaptive AFLP loci in the invasive populations of *M. micrantha* (Wang et al., 2012).

Chloroplasts are essential cellular organelles, which have a decisive effect on the growth, development, and biotic defense of plants (Nie et al., 2012). Tolerances of environmental stress, including drought, high salinity, extremes of temperature, heavy metals, and high light, are all known associated with the function of chloroplast (cp) genomes (Fitzgerald et al., 2011). For example, it has been revealed that high levels of chloroplast genomic diversity are important in allowing populations of weeping ricegrass to adapt to both warm and dry climatic conditions (Fitzgerald et al., 2011). Nonetheless, whether the cp genome is involved in the adaptation of invasive plants to novel environments remains unclear. Chloroplast DNA variation can be effectively detected by chloroplast simple sequence repeats (cpSSRs). These cpSSRs are uniparentally inherited and non-recombinant. They are particularly efficient for the detection of bottleneck effects, because the cp genome is haploid (Rodriguez et al., 2013). To complement the analysis of nuclear genome for understanding the response of *M. micrantha* to local edaphic conditions (Wang et al., 2012), we have identified variable cpSSRs and evaluated associations among haplotypes and soil characteristics.

In the present study, we have measured the soil composition and conducted a cpSSR assay for the invasive populations of *M. micrantha* in southern China. We focus to test the following hypotheses: (i) whether a negative correlation exists between the genetic diversity of a population and its geographic distance from the origin of introduction; (ii) are there any adaptive cpSSR loci that are significantly associated with soil attributes? This study will provide additional information to improve our ability to predict the invasion of *M. micrantha* in southern China and mitigate its impact.

## Materials and Methods

### Plant Materials and Soil Sampling

**FIGURE 1:**
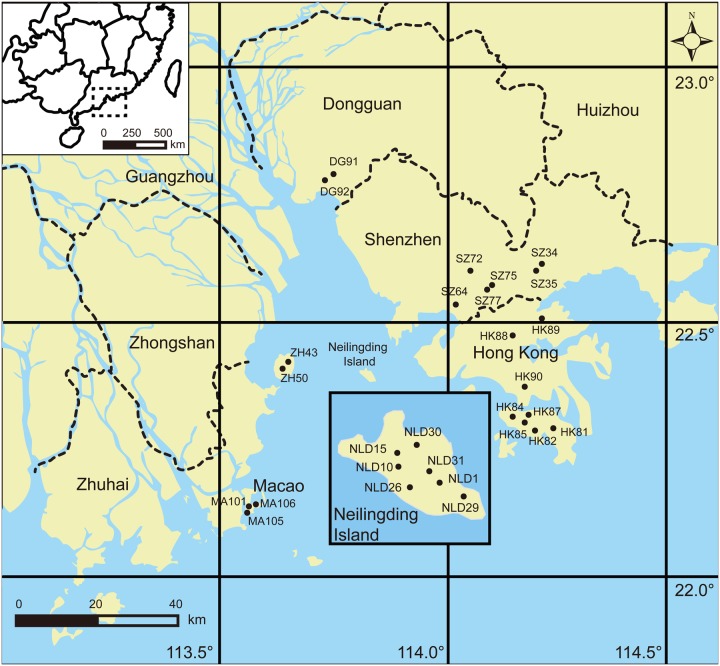
**Detailed sampling locations of *M. micrantha* in southern China shown by black solid points**.

Twenty-eight *M. micrantha* populations covering its entire introduced range in southern China were sampled as described by Wang et al. (2012). The plant materials were collected and processed as described in the previous study (Wang et al., 2012; **Figure 1**). To obtain environmental data, three soil samples per population were randomly selected and analyzed. Each soil sample, of about 0.5 g, was taken from a depth of 10–15 cm.

### Soil Composition Analysis

Soil samples were air dried and ground fine enough to pass through a 1.0 mm sieve. The measurement of soil pH was performed in suspension at a ratio of 2:5 (W/V) of soil to deionized water using a pH meter (PHSJ3F, INESA, Shanghai, China). Electrical conductivity was measured using a conductivity meter (model DDS-307A, INESA, Shanghai, China), soil moisture was determined by oven drying for 6 h at 105°C, and soil organic matter content was measured by the wet combustion method. Total nitrogen and total phosphate were digested with H_2_SO_4_ + HClO_4_ at a ratio of 5:1 (W/V) and then determined using the indophenol-blue and molybdenum-blue method, respectively. Total soil Al, Ca, Cu, Fe, K, Mg, Mn, Na, Si, and Zn concentrations were determined by using inductively coupled plasma atomic emission spectrometry (ICP-AES) after acid digestion.

### DNA Extraction and cpSSR Protocols

DNA extraction methods were presented in detail in a previous study (Wang et al., 2012). To optimize the cpSSR conditions, a total of 34 cp microsatellite loci were preliminarily screened. We chose eight polymorphic cp microsatellites (**Table 1**). Only cpSS 02 was an anonymous marker. It is unclear whether cpSS 02 is located in a coding or non-coding region of the cp genome. Polymerase chain reaction (PCR) was performed in a 20 μL final volume containing 1 × buffer, 25 Mm MgCl_2_, 10 Mm each of dNTP, 0.5 U *Taq* polymerase, 5 mm of each primer, and 100 ng template DNA. The PCR program was as follows: an initial denaturation of 5 min at 94°C, followed by 35 cycles of denaturation at 94°C for 60 s, annealing at 55°C for 60 s, and elongation at 72°C for 120 s, and a final elongation of 72°C for 5 min. PCRs were conducted on a PTC-100 Peltier Thermal Cycler (MJ Research). The PCR products were sized on an ABI 377 automated sequencer (Applied Biosystems) using ROX-500 as the size marker.

**Table 1:** Primer sequences for cpSSR analysis.

Number	Sequence 5′-3′	Reference
cpSS02	CTAACGATGCGGGTTCGATTC	Zhang X.Y. et al., 2004
	CCTATACCGAAGGTTTAGAAGACCTC	
RCt8	ATAGTCAAGAAAGAGGATCTAGAAT	Ishii and McCouch, 2000
	ACCGCGATTCAATAAGAGTA	
RCt9	ATAAGGTTATTCCCCGCTTACC	
	AAATTGGGGGAATTCGTACC	
WCt2	CTTATCTAATGACCCAGGACGG	Ishii et al., 2001
	CGAATTGGAAAGAATTCTGACC	
WCt11	TTTTATCTAGGCGGAAGAGTCC	
	TCATTTGGCTCTCACGCTC	
WCt12	CGATCCCTATGTAGAAAGCCC	
	AACGAAACCCCTTCTTACCG	
WCt14	TCAACAAGTGACTCGAACTGTG	
	CGTCATGGAATAGGTGTCTCA	
WCt22	GCAATAGTGTCCTTGCCCAT	
	ACCAAAATAGTTTCATTAGCTCCTG	


### Statistical Analysis

Raw fluorescent cpSSR data were collected and scored using software GeneScan 3.7 (Applied Biosystems) and genographer (version 1.6.0)^1^. GenALEx 6.3 was used to calculate population genetic parameters including percentage of polymorphic loci, observed number of alleles, effective number of alleles, Nei’s gene diversity, and Shannon’s Information index. The occurrence of null alleles in cpSSR data was determined using MICRO-CHECKER version 2.2.3.

Based on cpSSR data, we measured the population differentiation among and within populations using ARLEQUIN 3.0. Analysis of molecular variance (AMOVA) based on one thousand random permutations were performed at three levels: regional, among-population, and within-population. Also using the same software, isolation by distance, which is the correlation between geographical distance (km) and genetic differentiation among populations, was investigated using the Mantel test. The pairwise 𝜃^B^ estimates, which can be obtained by HICKORY v1.0, comprised the matrix of genetic differentiation. To investigate correlation between genetic variation and dissimilarity in soil composition, a partial Mantel test was also performed to evaluate dependence between these variables implemented in GenAlEx (9999 permutations), controlling for the effect of geographic distance.

We used SPAGeDi v. 1.3 (Hardy and Vekemans, 2002) to estimate Moran’s *I*, which quantifies the correlation between allelic frequency and spatial distance classes. The distance classes were 0.1, 0.2, 0.3, 0.4, 0.5, 0.6 and 0.7 km in order to obtain a high spatial resolution and to assure a sufficient number of individual pairs per distance class. Tests of significance were carried out against 9999 random permutations and 95% confidence intervals (CIs) were estimated.

Pearson correlation and linear regression analyses were performed to estimate each diversity parameter against the distance from Hong Kong Zoological and Botanical Gardens, the inferred original invasive site of *M. micrantha* in Southern China (Wang et al., 2003).

STRUCTURE 2.2.3 was used to further investigate the Bayesian clustering and assignment within the entire sample studied. Total 100 000 MCMC iterations and a 10 000 burn-in period were performed with admixture model to search for best number of clusters based on 30 independent runs. The maximum-likelihood estimate method suggested by Evanno et al. (2005) was applied to determine the number of clusters.

Linkage disequilibrium (LD) across loci may bias the estimation of the population genetic variation and differentiation (Volis et al., 2005). In order to remove the effects of LD, the extent of LD between all pairs of cpSSR loci was examined by using TASSEL (Trait Analysis by aSSociation, Evolution, and Linkage) 3.0 (Bradbury et al., 2007). The evaluated statistics included the standardized disequilibrium coefficient (*D’*) and the squared correlation coefficient (*r*^2^). Their significance was determined by a two-tailed Fisher’s exact test.

The program BOTTLENECK was used to test whether populations have suffered a bottleneck in the cp genome. The heterozygosity (Heq) expected at mutation-drift equilibrium was calculated based on both the stepwise mutation model (SMM) and the infinite allele model (IAM). The significance of heterozygosity excess was determined by the sign test.

We used two population genetic approaches to detect outlier loci, i.e., those that show a higher than expected differentiation between populations. First, the software Dfdist a frequentist method based on summary statistics of a symmetrical island model (i.e., drift-migration equilibrium). We used Dfdist to test for the neutrality of cpSSR markers. The 4 *N*μ parameter value was set to 0.04 in all simulations. All 28 populations were used for a global analysis of *M. micrantha.* The outlier threshold was delimited by more restrictive significance levels of 0.005. In addition, to cross-check the reliability of the outlier loci detected by Dfdist, we also ran Bayescan analyses. Bayescan was run with 10 pilot runs of length 5000, followed by 50,000 iterations each, 5000 sample sizes, and 20 thinning intervals. Generally, outliers identified by both Dfdist and Bayescan are likely to be truly adaptive regions of the genome.

Program GESTE 2.0, a hierarchical Bayesian method was used to analyze the relationship between genetic structure and environmental factors at each population site. *F*_ST_ values for each population and association of *F*_ST_ to environmental factors were estimated using a generalized linear model. The posterior probabilities for each model were provided using a reversible jump MCMC approach based on a sample size of 30,000. The model with the highest posterior probability was the one that best explained the data. We used a burn in of 100,000 iterations with 10 pilot runs of 5,000 iterations and a thinning interval of 50.

## Results

### Genetic Variation of cpSSR

Of the 34 chloroplast microsatellites screened, seven loci (RCt8, RCt9, WCt2, WCt11, WCt12, WCt14, and WCt22) were polymorphic and contributed to 39 alleles in *M. micrantha*. CpSS 02 was determined to be null alleles using the program Micro-Checker. We excluded this locus from further analyses. Genetic variation revealed with the cpSSR markers was high in *M. micrantha*. Across all of the populations, the percentage of polymorphic loci was 97.44%, and Nei’s gene diversity and Shannon’s Information Index were estimated to be 0.1686 and 0.2827, respectively.

At the population level, all but one measure (the number of polymorphic loci) of cpSSR genetic diversity were highest in population NLD26. Measures that were highest in NLD26 were: the observed number of alleles, the effective number of alleles, Nei’s gene diversity, and Shannon’s Information Index (**Table 2**). In contrast, all estimates except effective number of alleles and Nei’s gene diversity were lowest in population HK87 (**Table 2**). At the region level, all estimates except effective number of alleles showed that region NLD had the highest level of genetic diversity. No region was found to have the least genetic variation across a majority of the estimates calculated.

**Table 2:** Estimates of cpSSR genetic diversity and genetic bottleneck in chloroplast genome for *Mikania micrantha* populations from six introduced regions in southern China.

Population	Number of polymorphic loci	Percentage of polymorphic loci	Observed number of alleles	Effective number of alleles	Nei’s gene diversity	Shannon’s Information Index	SMM		IAM	
		
							He/Hd	*P*	He/Hd	*P*
NLD1	22	70.97	1.7097	1.385	0.2271	0.346	8/30	0.00002	9/29	0.00471
NLD10	18	64.29	1.6429	1.3656	0.2161	0.3265	7/31	0.00000	8/30	0.00174
NLD15	9	36	1.36	1.1469	0.0944	0.1514	6/32	0.00000	6/32	0.00009
NLD26	21	95.45	1.9545	1.6853	0.3794	0.5528	9/29	0.00008	11/27	0.02488
NLD29	11	44	1.44	1.234	0.1407	0.2152	6/32	0.00000	6/32	0.00010
NLD30	16	53.33	1.5333	1.2613	0.1583	0.2457	7/31	0.00000	8/30	0.00174
NLD31	15	57.69	1.5769	1.3088	0.1828	0.2793	6/32	0.00000	7/31	0.00037
NLD	36	97.3	1.973	1.2936	0.1928	0.3185	11/27	0.01041	13/25	0.20705
SZ34	17	56.67	1.5667	1.2315	0.15	0.2403	7/31	0.00000	7/31	0.00046
SZ35	11	44	1.44	1.1732	0.1117	0.1802	5/33	0.00000	6/32	0.00010
SZ64	19	63.33	1.6333	1.2874	0.1802	0.2833	8/30	0.00002	8/30	0.00126
SZ72	10	47.36	1.4762	1.2465	0.1498	0.2298	5/33	0.00000	6/32	0.00012
SZ75	19	55.88	1.5588	1.2449	0.1555	0.246	8/30	0.00002	8/30	0.00178
SZ77	18	60	1.6	1.3224	0.1963	0.2997	7/31	0.00000	8/30	0.00136
SZ	26	70.27	1.7027	1.234	0.1533	0.2507	9/29	0.00073	12/26	0.06624
ZH43	21	65.62	1.6562	1.2785	0.1772	0.282	7/31	0.00000	7/31	0.00044
ZH50	19	61.29	1.6129	1.2536	0.1626	0.2597	7/31	0.00000	7/31	0.00045
ZH	24	72.73	1.7273	1.2926	0.1911	0.3058	8/30	0.00006	12/26	0.13847
HK81	11	45.83	1.4583	1.2324	0.139	0.2145	7/31	0.00000	7/31	0.00046
HK82	13	52	1.52	1.2888	0.1694	0.2571	8/30	0.00002	9/29	0.00425
HK84	12	48	1.48	1.3215	0.1804	0.2654	8/30	0.00002	8/30	0.00139
HK85	19	59.38	1.5938	1.3093	0.1893	0.2905	10/28	0.00023	10/28	0.01197
HK87	8	33.33	1.3333	1.1572	0.0995	0.1553	6/32	0.00000	6/32	0.00010
HK88	17	58.62	1.5862	1.2501	0.1617	0.2569	8/30	0.00002	8/30	0.00163
HK89	15	53.57	1.5357	1.3157	0.1782	0.2675	8/30	0.00002	9/29	0.00468
HK90	14	45.16	1.4516	1.2294	0.1435	0.2215	8/30	0.00002	8/30	0.00129
HK	27	71.05	1.7105	1.2335	0.1452	0.2343	10/28	0.00612	10/28	0.02184
MA101	14	51.85	1.5185	1.3404	0.1936	0.286	8/30	0.00002	9/29	0.00421
MA105	12	46.15	1.4615	1.2461	0.1447	0.2216	7/31	0.00000	7/31	0.00040
MA106	9	42.86	1.4286	1.3316	0.1833	0.2642	7/31	0.00000	7/31	0.00037
MA	18	62.07	1.6207	1.3141	0.1885	0.2883	9/29	0.00074	12/26	0.01274
DG91	10	50	1.5	1.2562	0.155	0.2385	4/34	0.00000	5/33	0.00002
DG92	19	63.33	1.6333	1.3211	0.1993	0.3072	8/30	0.00001	8/30	0.00149
DG	21	47.74	1.6774	1.2352	0.159	0.2605	11/27	0.00254	11/27	0.06254
Total	38	97.44	1.9744	1.259	0.1686	0.2827	13/25	0.14421	13/25	0.35486


*Abbreviations: SMM: the stepwise mutation model. IAM: the infinite allele model.*

At the region level, *G*_ST_ values were 0.2625, 0.2172, 0.2150, 0.2061, 0.1510, and 0.0795 for regions NLD, MA, HK, SZ, ZH, and DG, respectively. That is, NLD had the most strongly differentiated populations, while DG populations were the least differentiated. Overall *G*_ST_ among the 28 populations was 0.2880.

Analysis of molecular variance indicated that most (84.53%) of the variance was attributable to the differences within populations, 7.85% was accounted for by differences among populations within regions, while 7.62% was attributable to differences among regions (**Table 3**). A random permutation test revealed that these variance partitions were all significant (*P* < 0.001). Wright’s *F*_ST_ was 0.1547.

**Table 3:** Analysis of molecular variance (AMOVA) for *Mikania micrantha* populations from six introduced regions in southern China.

	d.f.	Sum of squares	Variance components	Percentage of total variation	*P*	*F* statistics
Among regions	5	63.218	0.28893	7.62	<0.001	*F*_CT_ = 0.07621
Among populations within regions	22	109.806	0.29773	7.85	<0.001	*F*_SC_ = 0.08501
Within populations	140	448.667	3.20476	84.53	<0.001	*F*_ST_ = 0.15473


**FIGURE 2:**
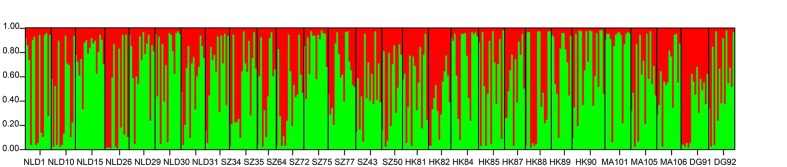
**Chloroplast genetic structure of *M. micrantha* populations based on structure.** Each color represents a different cluster. Black lines separate populations. For population codes see **Table 2**.

In the STRUCTURE analysis, the clustering level, *K* = 2, yielded the largest delta-*K*-value (Δ*K* = 136.77835). Both of the clusters exhibited substantial admixture, which is visualized in **Figure 2**.

**FIGURE 3:**
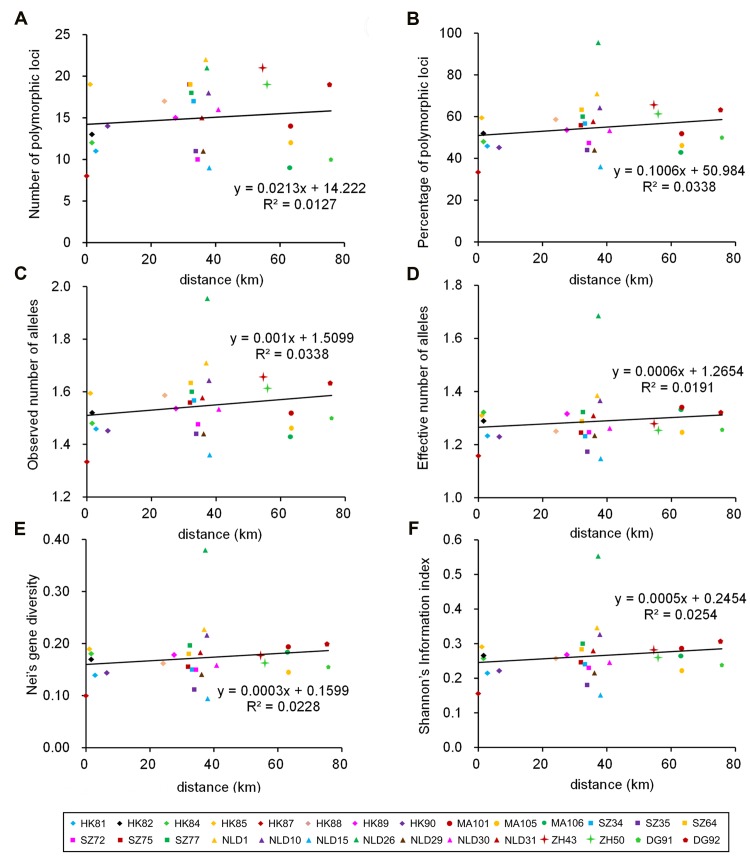
**Linear regressions between number of polymorphic loci **(A)**, percentage of polymorphic loci **(B)**, observed number of alleles **(C)**, effective number of alleles **(D)**, Nei’s gene diversity **(E)**, and Shannon’s information index **(F)** and the distance of sites from Hong Kong Zoological and Botanical Gardens and calculated over 39 variable loci (Pearson, *P* > 0.05)**.

The matrix of genetic distances among populations was not significantly correlated with the corresponding matrix of geographical distances (Mantel test; *r* = 0.0711, *P* = 0.293). That is, no evidence of “isolation by distance” was detected. Because genetic diversity is expected to decrease during the invasion of new regions, regression of diversity on geographic distance from an inferred ancestral source population would reveal a negative correlation coefficient. However, we found that each diversity parameter had no significant correlation with increasing distance from Hong Kong Zoological and Botanical Gardens (Pearson’s r: *P* > 0.05), which followed the linear regression models reported in **Figure 3**. From Hong Kong to Guangdong, the genetic diversity of *M. micrantha* decreased. However, genetic variation in NLD 26 reaches a maximum, then it gradually decreased in other areas. In addition, we also compared the dissimilarity of soil attributes among populations with their genetic distances with the Mantel test; there was no significant relation between these variables.

**FIGURE 4:**
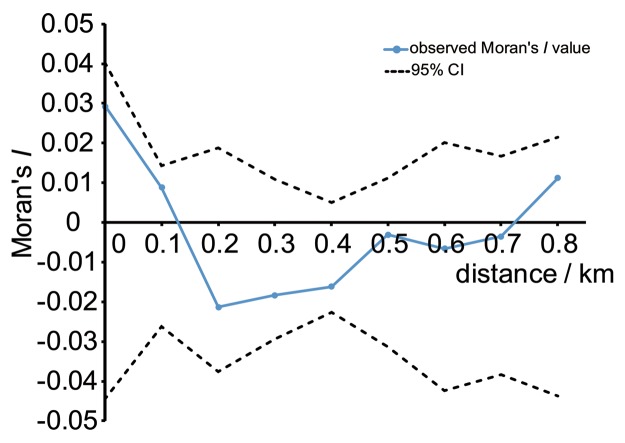
**Spatial autocorrelation analysis of genetic and geographical distance in *M. micrantha* using SPAGeDi.** Dashed lines indicate 95% confidence intervals, assuming the null hypothesis that no autocorrelation exists, based on 9999 permutations.

Moran’s *I* was insignificant for all distance classes, indicating that the overall spatial pattern of genetic variation is not differentiable from random across the entire study region (**Figure 4**). Similarly, at a finer spatial scale, no significant value of *I* was detected across different distance classes, ranging from 0 to 0.8 km (**Figure 4**).

### Linkage Disequilibrium and Bottleneck Test

All 39 cpSSR alleles genotyped on the 28 populations were tested for linkage disequilibrium (LD). No allelic pairs were found to be in significant LD based on *D*’ and *r*^2^ statistics (**Table 4**).

**Table 4:** The relationship between *D’*, as well as *r*^2^ and *P*-values among loci.

Locus	*D’*	*r*^2^	*P*
2 and 1	0.548872	9.68 × 10^-6^	0.1232
5 and 4	0.83783	0	0.4722
7 and 5	0.675676	4 × 10^-8^	0.30712
7 and 6	0.682119	0	0.375713
14 and 13	0.552795	3.66 × 10^-5^	0.305582
18 and 16	0.864734	0.000731	0.06254
19 and 16	1	3.1 × 10^-7^	0.127614
19 and 17	1	8.45 × 10^-5^	0.077584
19 and 18	0.671362	1 × 10^-8^	0.295399
20 and 17	0.668148	3 × 10^-8^	0.176116
20 and 18	0.785441	0.00026	0.079514
20 and 19	0.851459	5.5 × 10^-7^	0.142576


When either the SMM or the IAM was assumed, no significant excess of heterozygosity was detected for any population up to and including at the level of the entire range (**Table 2**).

### Detection of Signatures of Positive Selection

**FIGURE 5:**
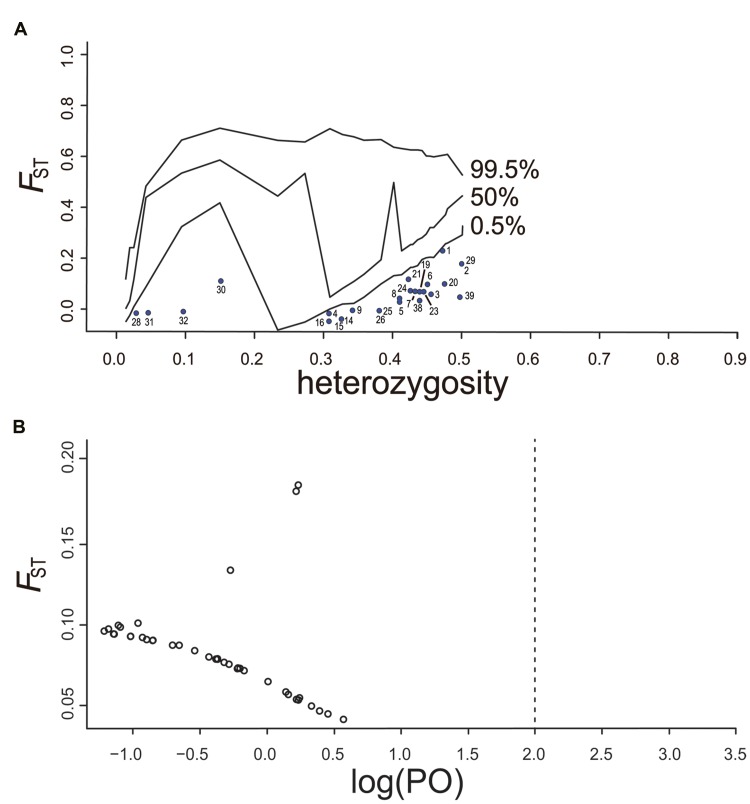
**(A)** Results of outlier detection from the simulation with Dfdist. Plots of *F*_ST_ value were against heterozygosity based on chloroplast SSR loci. The 0.5, 50, and 99.5% confidence intervals were corresponding to the lower, intermediate, and higher lines, respectively. No outlier locus under selection above the 99.5% line was detected in chloroplast genome. **(B)** Results of outlier loci from chloroplast SSR genomic scan by Bayescan. The vertical line showed the log_10_ of the posterior odds (PO), which provides evidence whether the locus is subject to selection or not. No outlier locus under selection in chloroplast genome was detected at the decisive threshold value (log_10_ PO = 2.0).

We used global *F*_ST_ simulations in an infinite-alleles model based on an island demographic model in Dfdist. We employed the 30% trimmed mean, which removes the highest and lowest 30% of *F*_ST_ values. This trimmed mean served as the target mean *F*_ST_ for coalescent simulations. In this study, no adaptive loci were identified at the 99.5% confidence level (**Figure 5A**). Only loci under negative selection were detected.

We adopted log_10_ PO > 2.0 as a threshold for decisive evidence for accepting a model under selection, which corresponded to a posterior probability greater than 0.91. No locus was found to exhibit a divergence pattern that deviated from neutral expectation (**Figure 5B**).

### Measurement of Soil Components

Site pH values ranged from 4.07 to 8.13, and organic matter content ranged from 0.70 to 35.65%. Si concentration varied from 0.02 to 2.68 mg/g. In terms of metal concentrations, Mg, Zn, and Al concentrations varied greatly, ranging from 0.048 to 63.9 mg/g, 0.0039 to 0.067 mg/g, and 0.35 to 16.80 mg/g, respectively. Details of soil components were showed in the supplementary Data Sheet.

### Association with Environmental Variables

GESTE analyses were carried out on all cpSSR loci and environmental variables. Generalized linear models were run using all seven non-metal factors (soil pH, electrical conductivity, fresh water content in soil, air dried soil water content, soil organic matter, total nitrogen, and total phosphate), resulting in a total of 128 models. For 10 metal factors, a total of 1024 models were produced. Mg was found to have the highest probability model, 513 (*Pr* = 0.00390). Due to the fact that no adaptive loci were identified in the cp genome, Mg appeared to have only a genome-wide effect instead of being a selective factor.

## Discussion

Our results showed that *M. micrantha* in southern China possesses relatively high cpSSR variation and differentiation (Wright, 1978; Powell et al., 1995; Hamrick and Godt, 1996). The genetic diversity is highest in NLD in Shenzhen. Genetic diversity is expected to decrease from the origin of introduction to newly invaded areas (Fontaine et al., 2013). However, *M. micrantha* chloroplast variation did not conform to this hypothesis. Compared to other regions, the population genetic variation at Hong Kong Zoological and Botanical Gardens, the site documented as the origin of introduction, is the lowest. It seems that with naturalization in southern China, the genetic variation of *M. micrantha* has gradually increased in the novel invasive areas. The Mantel test detected no correlation between genetic and geographic distances. Moreover, the Moran’s *I* correlogram also showed no spatial genetic structure. Both analyses suggest that the genetic variation is randomly distributed across the introduced populations of *M. micrantha*. Similar results have been obtained in an ISSR analysis of *M. micrantha* (Wang et al., 2008).

The pattern of population genetic variation observed here may be closely linked with the ecological characteristics of *M. micrantha*. The weed produces enormous numbers of small and light seeds (1.7 × 10^5^ /m^2^; Zhang L.Y. et al., 2004). Although its seed dispersal takes place by wind, water and animals, dispersal by wind is thought to be the chief means of invasion into disturbed environments (Zhang L.Y. et al., 2004). In particular, violent winds have the potential to uplift and transport the seeds several hundred kilometers away (Zhang L.Y. et al., 2004). We infer that extensive gene flow mediated by wind-dispersed seeds is likely the primary factor causing the random distribution of genetic variation across the introduced region.

Besides seeds, *M. micrantha* can also reproduce vegetatively by producing shoots from stem fragments and rosettes (Zhang L.Y. et al., 2004). Vegetative reproduction tends to increase the occurrence of patches of genetically identical individuals (ramets), which will yield significant positive values of Moran’s *I* for small distance classes in the correlogram. However, this is not the case for *M. micrantha* (**Figure 3**). Moreover, lack of spatial genetic structure also indicates that populations of *M. micrantha* are not subdivided into local demes or subpopulations that are consisted of interbreeding, closely related plants. These results, which suggest that dispersal and establishment via seed prevail over vegetative expansion, may be helpful for developing an efficient control program.

Invasive species are generally characterized with genetic bottlenecks (Garbelotto et al., 2013). In the previous, we have detected evidence for bottlenecks in the nuclear genome of *M. micrantha* (Wang et al., 2012). Compared with nuclear markers, cpSSRs are particularly efficient for the detection of bottleneck effects due to their haploid nature (Rodriguez et al., 2013). Surprisingly, however, no bottleneck was found in the *M. micrantha* cp genome, possibly attributing to the lack of the accumulation of mutations or recombinations (Garbelotto et al., 2013). In addition, gene flow through seed dispersal, which causes mixing of divergent chloroplasts, can also erase bottleneck effects. The lack of linkage disequilibrium in the *M. micrantha* populations may also be associated with the mixing of chloroplasts.

We also performed a scan of the *M. micrantha* cp genome by using cpSSRs. No adaptive loci were detected in this study. By contrast, 14 adaptive loci were identified in the previous AFLP genome scan (Wang et al., 2012). It seems that the local adaptation of *M. micrantha* is driven by nuclear rather than cp genome since its introduction to southern China. The likely underlying causes are as follows. First, angiosperm cp genomes are evolutionarily conserved and have lower rates of nucleotide substitution than nuclear genomes (Guisinger et al., 2008). Asteraceae cp genomes are known especially conservative (Nie et al., 2012). Their low levels of substitution input may limit the adaptability, although this supposition remains controversial (Harris et al., 2012; Bock et al., 2014). Second, most chloroplast genes of photosynthetic plants undergo strong negative rather than positive selection due to functional constraints (Guisinger et al., 2008). Adaptive evolution has only been detected in a few chloroplast genes (Erixon and Oxelman, 2008; Iida et al., 2009; Sen et al., 2012). Third, chloroplast is semi-autonomous. A chloroplast gene-encoding subunit often needs to assemble with a nuclear gene-encoding counterpart to form a functional complex (Kellogg and Juliano, 1997). Thus, if a substitution happens to occur in a chloroplast gene, a compensatory substitution(s) in its interacting nuclear gene would be needed to maintain the function of the whole complex (Jansen et al., 2007). This will impose extra selective pressure to restrain the occurrence of adaptive evolution. To better understand the issue, we have sequenced the entire cp genome from multiple accessions of *M. micrantha* (Huang et al., 2016).

In this study, we observed that soil Magnesium (Mg), instead of just being a selective factor, has a genome-wide effect on cpDNA. Mg is an essential element for plant growth and development, whose biological functions include key roles in photosynthesis, protein synthesis, and nucleotide metabolism (Gransee and Führs, 2013). In particular, Mg^2+^ acts as an important signal in the regulation of key enzymes involved in the fixation of carbon in chloroplasts (Shaul, 2002). Small variation in Mg^2+^ levels at the cytosol and the chloroplast can strongly affect the activity of photosynthetic enzymes (Shaul, 2002). More importantly, Mg is the central atom in the chlorophyll molecule. Its deficiency will result in mild to severe etiolation in leaves (Gransee and Führs, 2013). Our finding of the effect of Mg on the *M. micrantha* cp genome highlights the association between soil Mg and the successful invasion of the weed in southern China.

## Conclusion

The present study is the first to report on chloroplast genomic diversity of the invasive weed *M. micrantha*. High genetic variability and differentiation, no linkage disequilibrium, and no severe bottlenecks were observed in the introduced populations of *M. micrantha* by using cpSSRs. Each genetic diversity parameter showed no significant correlation with increasing distance from the origin of introduction. Soil appeared not associated with the cpDNA variation of *M. micrantha.* Our results provide a framework for further exploring the cp genome evolution of *M. micrantha*.

## Author Contributions

TW designed and performed the experiments, and wrote the manuscript; ZW conducted data analysis and checked English grammar; GC performed the cpSSR experiment and soil analysis; CW performed data analysis; YS contributed to the supervision of the work and wrote the manuscript. All authors read and approved the final version of the manuscript.

## Conflict of Interest Statement

The authors declare that the research was conducted in the absence of any commercial or financial relationships that could be construed as a potential conflict of interest.

## Footnotes

1 http://genographer.software.informer.com/download/

## References

[B1] Alberto F. Niort J. Derory J. Lepais O. Vitalis R. Galop D. (2010). Population differentiation of sessile oak at the altitudinal front of migration in the French Pyrenees. *Mol. Ecol.* 19 2626–2639. 10.1111/j.1365-294X.2010.04631.x 20561196

[B2] Anacker B. L. Whittal J. B. Goldberg E. E. Harrison S. P. (2011). Origins and consequences of serpentine endemism in the California flora. *Evolution* 65 365–376. 10.1111/j.1558-5646.2010.01114.x 20812977

[B3] Austerlitz F. Jung M. B. Godelle B. Gouyon P. H. (1997). Evolution of coalescence times, genetic diversity and structure during colonization. *Theor. Popul. Biol.* 51 148–164. 10.1006/tpbi.1997.1302

[B4] Bock D. G. Andrew R. L. Rieseberg L. H. (2014). On the adaptive value of cytoplasmic genomes in plants. *Mol. Ecol.* 23 4899–4911. 10.1111/mec.12920 25223488

[B5] Bradbury P. J. Zhang Z. Kroon D. E. Casstevens T. M. Ramdoss Y. Buckler E. S. (2007). TASSEL: software for association mapping of complex traits in diverse samples. *Bioinformatics* 23 2633–2635. 10.1093/bioinformatics/btm308 17586829

[B6] Dlugosch K. M. Parker I. M. (2008). Founding events in species invasions: genetic variation, adaptive evolution, and the role of multiple introductions. *Mol. Ecol.* 17 431–449. 10.1111/j.1365-294X.2007.03538.x 17908213

[B7] Erixon P. Oxelman B. (2008). Whole-gene positive selection, elevated synonymous substitution rates, duplication, and indel evolution of the chloroplast *clpP1* gene. *PLoS ONE* 3:e1386 10.1371/journal.pone.0001386 PMC2148103 18167545

[B8] Evanno G. Regnaut S. Goudet J. (2005). Detecting the number of clusters of individuals using the software STRUCTURE: a simulation study. *Mol. Ecol.* 14 2611–2620. 10.1111/j.1365-294X.2005.02553.x 15969739

[B9] Fitzgerald T. L. Shapter F. M. McDonald S. Waters D. L. E. Chivers L. H. Drenth A. (2011). Genome diversity in wild grasses under environmental stress. *Proc. Natl. Acad. Sci. U.S.A.* 108 21140–21145. 10.1073/pnas.1115203108 22173638 PMC3248542

[B10] Fitzpatrick B. M. Fordyce J. A. Niemiller M. L. Graham R. R. (2012). What can DNA tell us about biological invasions? *Biol. Invasions* 14 245–253. 10.1111/mec.13307

[B11] Fontaine M. C. Austerlitz F. Giraud T. Labbé F. Papura D. Richard-Cervera S. (2013). Genetic signature of a range expansion and leap-frog event after the recent invasion of Europe by the grapevine downy mildew pathogen *Plasmopara viticola*. *Mol. Ecol.* 22 2771–2786. 10.1111/mec.12293 23506060

[B12] Garbelotto M. Guglielmo F. Mascheretti S. Croucher P. J. P. Gonthier P. (2013). Population genetic analyses provide insights on the introduction pathway and spread patterns of the North American forest pathogen *Heterobasidion irregulare* in Italy. *Mol. Ecol.* 22 4855–4869. 10.1111/mec.12452 24033583

[B13] Gransee A. Führs H. (2013). Magnesium mobility in soils as a challenge for soil and plant analysis, magnesium fertilization and root uptake under adverse growth conditions. *Plant Soil* 368 5–21. 10.1007/s11104-012-1567-y

[B14] Guisinger M. M. Kuehl J. V. Boore J. L. Jansen R. K. (2008). Genome-wide analyses of Geraniaceae plastid DNA reveal unprecedented patterns of increased nucleotide substitutions. *Proc. Natl. Acad. Sci. U.S.A.* 105 18424–18429. 10.1073/pnas.0806759105 19011103 PMC2587588

[B15] Hamrick J. L. Godt M. J. W. (1996). Effects of life history traits on genetic diversity in plant species. *Philos. Trans. R. Soc. Lond. B* 351 1291–1298. 10.1098/rstb.1996.0112

[B16] Hancock A. M. Brachi B. Faure N. Horton M. W. Jarymowycz L. B. Sperone F. G. (2011). Adaptation to climate across the *Arabidopsis thaliana* genome. *Science* 334 83–86. 10.1126/science.1209244 21980108

[B17] Hardy O. J. Vekemans X. (2002). SPAGeDi: a versatile computer program to analyze spatial genetic structure at the individual or population levels. *Mol. Ecol. Notes* 2 618–620. 10.1046/j.1471-8286.2002.00305.x

[B18] Harris C. J. Dormontt E. E. Le Roux J. J. Lowe A. Leishman M. R. (2012). No consistent association between changes in genetic diversity and adaptive responses of Australian acacias in novel ranges. *Evol. Ecol.* 26 1345–1360. 10.1007/s10682-012-9570-6

[B19] Holm L. G. Plucknett D. L. Pancho J. V. Herberger J. P. (1977). *The World’s Worst Weeds: Distribution and Biology*. Honolulu: East-West Center and University Press of Hawaii.

[B20] Huang L. Wang Z. Wang T. Su Y. J. (2016). The complete chloroplast genome sequence of *Mikania micrantha* (Asteraceae), a noxious invasive weed to South China. *Mitochondrial DNA B Resour.* 1 603–604. 10.1080/23802359.2016.1209090 PMC7800490 33473567

[B21] Huang Z. L. Cao H. L. Liang X. D. Ye W. H. Feng H. L. Cai C. X. (2000). The growth and damaging effect of *Mikania micrantha* in different habitats. *J. Trop. Subtrop. Bot.* 8 131–138.

[B22] Iida S. Miyagi A. Aoki S. Ito M. Kadono Y. Kosuge K. (2009). Molecular adaptation of *rbcL* in the heterophyllous aquatic plant *Potamogeton*. *PLoS ONE* 4:e4633 10.1371/journal.pone.0004633 PMC2646136 19247501

[B23] Ishii T. McCouch S. R. (2000). Microsatellites and microsynteny in the chloroplast genomes of Oryza and eight other Gramineae species. *Theor. Appl. Genet.* 100 1257–1266. 10.1007/s001220051432

[B24] Ishii T. Mori N. Ogihara Y. (2001). Evaluation of allelic diversity at chloroplast microsatellite loci among common wheat and its ancestral species. *Theor. Appl. Genet.* 103 896–904. 10.1007/s001220100715

[B25] Kellogg E. Juliano N. (1997). The structure and function of Rubisco and their implications for systematic studies. *Am. J. Bot.* 84 413–428. 10.2307/2446015 21708595

[B26] Jansen R. K. Cai Z. Raubeson L. A. Daniell H. dePamphilis C. W. Leebens-Mack J. (2007). Analysis of 81 genes from 64 plastid genomes resolves relationships in angiosperms and identifies genome-scale evolutionary patterns. *Proc. Natl. Acad. Sci. U.S.A*. 104 19369–19374. 10.1073/pnas.0709121104 18048330 PMC2148296

[B27] Liu X. W. Zhou Y. L. Qi C. M. Li Y. Wang Q. X. Guo M. X. (2012). Effects of *Mikania micrantha* invasion on soil nutrient contents and enzyme activities. *Ecol. Environ. Sci.* 21 1960–1965.

[B28] Misiewicz T. M. Fine P. V. A. (2014). Evidence for ecological divergence across a mosaic of soil types in an Amazonian tropical tree: *Protium* subserratum (Burseraceae). *Mol. Ecol.* 23 2543–2558. 10.1111/mec.12746 24703227

[B29] Nie X. J. Lv S. Z. Zhang Y. X. Du X. H. Wang L. Biradar S. S. (2012). Complete chloroplast genome sequence of a major invasive species, crofton weed (*Ageratina adenophora*). *PLoS ONE* 7:e36869 10.1371/journal.pone.0036869 PMC3350484 22606302

[B30] Powell W. Morgante M. McDevitt R. Rafalaski J. A. (1995). Polymorphic simple sequence repeat regions in chloroplast genomes: applications to the population genetics of pines. *Proc. Natl. Acad. Sci. U.S.A.* 92 7759–7763. 10.1073/pnas.92.17.7759 7644491 PMC41225

[B31] Prugnolle F. Manica A. Balloux F. (2005). Geography predicts neutral genetic diversity of human populations. *Curr. Biol.* 15 159–160. 10.1016/j.cub.2005.02.038 PMC1800886 15753023

[B32] Rodriguez M. Rau D. Angioi S. A. Bellucci E. Bitocchi E. Nanni L. (2013). European *Phaseolus coccineus* L. landraces: population structure and adaptation, as revealed by cpSSRs and phenotypic analyses. *PLoS ONE* 8:e57337 10.1371/journal.pone.0057337 PMC3579852 23451209

[B33] Sakata Y. Itami J. Isagi Y. Ohgushi T. (2015). Multiple and mass introductions from limited origins: genetic diversity and structure of *Solidago altissima* in the native and invaded range. *J. Plant Res.* 128 909–921. 10.1007/s10265-015-0753-4 26423999

[B34] Sen L. Fares M. A. Su Y. J. Wang T. (2012). Molecular evolution of *psbA* gene in ferns: unraveling selective pressure and co-evolutionary pattern. *BMC Evol. Biol.* 12:145 10.1186/1471-2148-12-145 PMC3499216 22899792

[B35] Shaul O. (2002). Magnesium transport and function in plants: the tip of the iceberg. *Biometals* 15 309–323. 10.1023/A:1016091118585 12206396

[B36] Volis S. Yakubov B. Shulgina I. Ward D. Mendlinger S. (2005). Distinguishing adaptive from nonadaptive genetic differentiation: comparison of QST and FST at two spatial scales. *Heredity* 95 466–475. 10.1038/sj.hdy.6800745 16189543

[B37] Wang B. S. Liao W. B. Zan Q. J. Li M. G. Zhou X. Y. Gao S. H. (2003). The spreads of *Mikania micrantha* in China. *Acta Sci. Nat. Univ. Sunyatseni* 42 47–50.

[B38] Wang T. Chen G. P. Zan Q. J. Wang C. B. Su Y. J. (2012). AFLP genome scan to detect genetic structure and candidate loci under selection for local adaptation of the invasive weed *Mikania micrantha*. *PLoS ONE* 7:e41310 10.1371/journal.pone.0041310 PMC3400595 22829939

[B39] Wang T. Su Y. J. Chen G. P. (2008). Population genetic variation and structure of the invasive weed *Mikania micrantha* in southern China: consequences of rapid range expansion. *J. Hered.* 99 22–33. 10.1093/jhered/esm080 17906304

[B40] Wright S. (1978). *Evolution and the Genetics of Populations: Variability Within, and Among Natural Populations*, Vol. 4 Chicago, IL: University of ChicagoPress, 79

[B41] Zhang L. Y. Ye W. H. Cao H. L. Feng H. L. (2004). *Mikania micrantha* H. B. K. in China – an overview. *Weed Res.* 44 42–49.

[B42] Zhang X. Y. Shiraishi S. Huang M. R. (2004). Analysis of genetic structure in population of *Larix Kaempferi* by chloroplast SSR markers. *Hereditas* 26 486–490. 15640046

